# Qualitative analysis of the coordination of major system change within the Colombian health system in response to COVID-19: study protocol

**DOI:** 10.1186/s43058-020-00063-z

**Published:** 2020-09-15

**Authors:** Simon Turner, Natalia Niño

**Affiliations:** School of Management, University of los Andes, Bogotá, Colombia

**Keywords:** Covid-19, Major system change, Evidence use, Adaptation, Decision-making, Colombia

## Abstract

**Background:**

Coronavirus (COVID-19) is posing a major and unprecedented challenge to health service planning and delivery across health systems internationally. This nationally funded study is analysing the response of the Colombian health system to the COVID-19 pandemic, drawing on qualitative case studies of three local health systems within the country. The approach will be informed by the concept of ‘major system change’—or coordinated change among a variety of healthcare organizations and other relevant stakeholders— to identify processes that both enable and inhibit adaptation of health services to the challenges presented by COVID-19. The study will collect information on capacity ‘bottlenecks’ as well as successful practices and forms of innovation that have emerged locally, which have the potential for being ‘scaled up’ across Colombia’s health services.

**Methods/design:**

This qualitative study will be undertaken in two phases. In the first, up to 30 stakeholder interviews will be conducted to ascertain immediate challenges and opportunities for improvement in response to COVID-19 that can be shared in a timely way with health service leaders to inform health service planning. The stakeholders will include planning, provider and intermediary organizations within the health system at the national level. In the second, up to 60 further interviews will be conducted to develop in-depth case studies of three local health systems at the metropolitan area level within Colombia. The interview data will be supplemented with documentary analysis and, where feasible, non-participant observation of planning meetings.

**Discussion:**

The study’s findings will aid evaluation of the relevance of the concept of major system change in a context of ‘crisis’ decision-making and contribute to international lessons on improving health systems’ capacity to respond to COVID-19 and future pandemics. Study findings will be shared among various stakeholders in the Colombian healthcare system in a formative and timely way in order to inform healthcare planning in response to COVID-19 and future pandemics. Conducting the study at a time of COVID-19 raises a number of practical issues (including physical distancing and pressure on health services) which have been anticipated in the study design and research team’s ways of working.

Contributions to the literature

- This study will explore the relevance of the concept of major system change, or coordinated improvements involving multiple stakeholders, in response to the COVID-19 pandemic in Colombia.
- Major system change has been applied to the study of longer-term planning of service change, but little is known about its relevance in a context of ‘crisis’ decision-making on, and fluidity of the evidence for, implementing change in the time of COVID-19.
- Through local health system case studies, this study will identify capacity ‘bottlenecks’ and novel practices, relationships and innovation that have emerged locally, which have the potential for replication and ‘scaling up’ across Colombia’s health services.

## Background

Coronavirus (COVID-19) is posing a major and unprecedented challenge to health service planning and delivery across health systems internationally [1]. Early evidence from Asian economies (Hong Kong, Singapore and Japan) coping with COVID-19 suggests that ‘integration of services in the health system and across other sectors amplifies the ability to absorb and adapt to shock’ [2]. However, local evidence is needed from other health systems internationally in order to examine the relevance of this hypothesis and to implement this and other organizational lessons for responding to COVID-19. Specific organizational problems need to be addressed to improve service integration at the operational and governance levels. These include developing the capacity, safety and quality of services to respond to demand from critically ill and other types of patient affected by COVID-19, directly or indirectly (service level), and coordinating a variety of individual health service organizations, municipalities and other stakeholders in the adaptive planning of services locally and nationally (governance level).

Colombia, an upper-middle-income country in Latin America, faces particular challenges in coordinating its response to system-wide problems like COVID-19. These relate to the antecedent conditions of the health system at the time of experiencing the pandemic. Although recent regulatory developments in Colombia promote the strengthening of leadership for population health and system change [3–5], and the need for multi-disciplinary and multi-sectoral care is recognized (Law 1438/2011), there remains a lack of system leadership and coordination [6]. Thus, enabling integration processes to create the ‘adaptive capacity’ [2] of suggested importance to Asian countries’ response to COVID-19 may be particularly challenging and will require active intervention and learning processes.

This study will examine the response of Colombia’s health system to COVID-19, both nationally and through local case studies, and provide timely, formative feedback to policymakers and health service leaders to inform health system planning. To generate such evidence, the study employs a multi-level, qualitative investigation of the national response of Colombia’s health system to COVID-19 and includes in-depth case studies of three major cities in Colombia—and governance relationships with the wider departments (states) in which they are situated—in order to trace local variation in responses. The study’s findings will be useful for health professionals responsible for leading health service delivery as well as those actors leading the formulation and implementation of public health policies.

The changes required by health systems to address the challenges posed by COVID-19 will be analysed using a social science concept, ‘major system change’ (MSC), that has been used to examine other forms of service-wide change (e.g. stroke service centralization) [7, 8]. MSC in health care involves coordinated change across multiple organizations, including providers and purchasers of services, across a metropolitan area or region with the aim of improving services across an entire geographical area [9]. There is an emerging literature on barriers and enablers of change involving multiple organizations, some of which have been distilled into a framework of ‘simple rules’ for guiding improvement work [7, 9]. These include involving stakeholders inside and outside the health service, including patients and communities, combining top-down and bottom-up leadership, creating feedback loops and learning from history or past experiences.

However, little is known about how navigating major system change is influenced by a context of ‘crisis’ decision-making, characterized by time pressure, complexity and uncertainty [10], as well as potential challenges with making sense of the fluidity of the evidence base for, and resources dependencies within health systems to, implement change in the time of COVID-19. This study will take account of how evidence informs decision-making on MSC during a time of ‘crisis’, including the availability, type and sources of evidence used; the nature of stakeholder involvement; and the ways in which such evidence is incorporated into health system planning [11] relative to examples of MSC with longer-term planning.

The project will address the following research question: ‘which organizational processes are enabling and constraining the response of health services in Colombia to COVID-19, both nationally and within local health systems, and what lessons for coordinating MSC can be drawn for informing the response to COVID-19 and future pandemics?’

This research question will be answered by meeting the following objectives:

1. To characterize, from the experiences of key actors of the health system at the national and local level, the strategies and organizational resources (including human resources and infrastructure) used to respond to the COVID-19 by each of the local health systems chosen for the study.

2. To identify critical coordination issues or ‘bottlenecks’ that are constraining the capacity of the selected local health systems to respond to the pandemic as well as factors that facilitate the response.

3. To analyse local examples of promising innovations and practices of coordination between actors that promote responsiveness to COVID-19 and have potential for scaling up more widely across health systems and/or integrated into healthcare guidelines or protocols.

4. To establish mechanisms for sharing lessons and experiences of the crisis in a timely fashion with health system leaders and other key stakeholders to inform health service planning in relation to COVID-19 and future pandemics of national and international origin.

The methods that will be used for generating evidence in relation to these objectives are outlined below.

## Methods/design

The study design will be informed by social science theory and research methods and adopt a qualitative, case study design [12]. Conceptually, the research will be informed by the theoretical frameworks of the social sciences that explore health services, and their integration, as ‘systems’ [13], MSC in health systems [7, 9] and care and management in relation to health emergencies [14, 15]. The study will be qualitative in order to give special attention to the narratives and the experience of the actors who have been directly involved in responding to the pandemic, at multiple levels of Colombia’s health system. The evidence developed will be drawn from stakeholder scoping discussions or interviews, analysis of secondary data sources and in-depth qualitative case studies of how three Colombian cities, including their departmental relationships, are responding to COVID-19. A qualitative approach was chosen due to, first, the exploratory or scoping nature of this study (i.e. the key priorities and research themes facing the health system in response to COVID-19 will be uncovered as data is collected) and, second, because of the need to gain the views and experiences of a range of stakeholders that can be uncovered most effectively through open-ended conversation or dialogue [16].

**Fig. 1:**
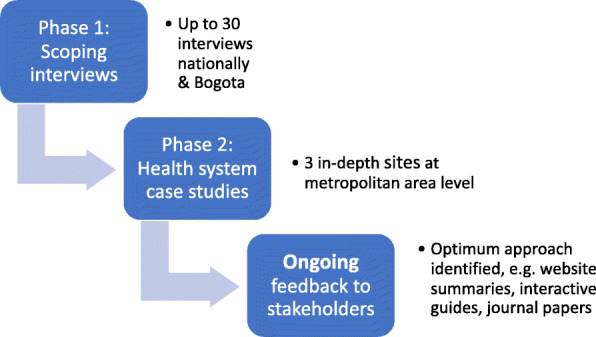
Phased study design and workplan

The study design involves two main phases and will establish mechanisms for providing timely, formative feedback to health system stakeholders (Fig. 1).

### Phase 1: scoping discussions or interviews

The aim of this rapid, initial phase is to acquire and share immediate challenges and opportunities for improvement in response to COVID-19 that can be shared in a timely way with health service leaders to inform immediate health service planning. This phase will focus on national level actors in Colombia, and the response of the health system to the pandemic in Bogotá, Colombia’s capital city. Bogotá, Colombia’s largest city with a population of 7.4 million, was chosen because it hosts all of the national governmental bodies that design the guidelines to respond to COVID-19 including the Ministry of Health, the Ministry of Science, Technology and Innovation and the National Institute of Health. Bogotá also centralizes many of the universities and national scientific associations and committees that are assessing the national response to the emergency. In addition, Bogotá was chosen given that it is the city that concentrates the highest number of patients positive for COVID-19 in the country (to 1 July 2020, Bogotá had 30,017 or 31% of the 97,846 cases across the country); this has been the trend since the emergency was declared in the country early in March 2020 [17]. Up to thirty scoping discussions will be conducted virtually in relation to Bogota either by telephone or using online applications (e.g. Microsoft Teams). The stakeholders will be selected in order to sample a range of planning, provider and intermediary organizations engaged in responding to COVID-19.

### Phase 2: qualitative case studies of health systems in Colombia

Using the research priorities and themes identified in phase 1 of the study, a deeper set of qualitative case studies of three health systems’ responses to COVID-19 in Colombia will be undertaken. This will cover the metropolitan area of Bogotá, developing the interview data acquired in phase 1, and two other local health systems within Colombia in order to compare and contrast health service needs and responses across different geographical territories of the country.

### Ongoing: identifying mechanisms for providing timely and formative feedback

The research will include, from the outset, investigation of the optimal mechanisms for the communication of emerging findings from the research study to maximize their impact on policy and practice in relation to COVID-19, as achieving impact is recognized as the driving purpose of the study. During the initial scoping discussions with stakeholders, their preferences for sharing feedback from the study (both content and approach) will be ascertained and collated. Thus, we will seek to share findings in a timely way, and as widely as possible with stakeholders and planners of local health systems, and nationally, in order to maximize their potential impact. This consultative approach will draw on and develop approaches used in previous research [18, 19] and principles of knowledge mobilization concerning the tailoring of findings to stakeholder audiences [20].

### Identifying relevant stakeholders

The stakeholders in phase 1 of the study will be limited to national-level interviewees and those relating to Bogotá’s health system in order to provide coherence across the interviews (i.e. all participants will be referring to a common health system across a metropolitan area) and to support the acquisition of contacts to interview across the metropolitan area (i.e. convenience and snowball sampling). Organizations, and potential interviewees within them, will be selected on the basis of those who have first-hand awareness of the situation facing the health system, nationally and in the three localities. This includes relevant actors in terms of health service providers, as well as key entities in the design of guidelines and planning of actions in public health. Once this network is identified, we will prioritize the most relevant organizations in responding to COVID-19 through stakeholder analysis. In these selected cases, we will carry out interviews with key actors within each organization who can give us a general and comprehensive vision of the way in which, from their specific role, they have responded to the emergency (for example, representatives of the local health secretary, hospital/clinic managers, leaders of scientific committees, representatives of the risk management unit).

We understand that access to these spaces in current circumstances represents a challenge and, for this reason, during the first phase of our project we will be in contact with key bodies nationally and locally that have links to the relevant communities of practice (e.g. Ministry of Science, Technology and Innovation, Ministry of Health and Social Protection and the National Institute of Health). The aim is to work closely with these entities and seek their support in negotiating access issues by providing official letters supporting the project, making suggestions of key organizations to be contacted and providing contact details of individuals in the organizations. Potential interviewees will be also be identified through informal discussion with colleagues in the School of Management, and other faculties including Government and Medicine, which have links to the relevant communities of practice within the health services and local government, including hospitals linked to the medical school.

### Study context and case selection

While recent interest in the theory and practice of MSC has grown [7–9, 21–23], there is a lack of research on the navigation of MSC within health systems in low- and middle-income countries (LMIC). Little is known about the professional and organizational processes shaping approaches to, and implementation of, change in LMIC in Latin America. Studying coordinated change in the LMIC context is important because such countries are likely to face particular resource pressures and technical constraints, e.g. on public health knowledge [5]. Colombia is an upper-middle-income country, and OECD member, with a Gross National Income (GNI) per capita of $6931 [24]. Colombia has a social health insurance model, funded through general taxation and payroll deductible contributions [25]. In 1992, the passing of Law 100 introduced compulsory health insurance for formal sector workers (and their families), while those working in the informal sector or unemployed would be covered by the lower cost ‘subsidized insurance scheme’. Patients choose an insurer to purchase services from public or private providers on behalf of their patients. Providers compete to deliver services to insurers who pay for services (so-called managed competition) [25].

Healthcare in Colombia is organized into networks of providers (Law 100/1993; Law 1122/2007), mainly private and some publicly owned, with primary care the entry point and coordinator of patient care. The territorial entities (departments, districts and municipalities) are responsible for public health activities, collective health programmes and health promotion. In relation to the population using the publicly subsidized regime, and the uninsured (4% of the population), the territorial entities contract services from publicly owned healthcare providers (these are social state enterprises). By 2012, the subsidized scheme covered 48.1% of the population [26]. Since 2015, both schemes have provided access to an equal range of services; however, judiciary intervention needed to address barriers to access to services is high [25]. Barriers to access are a particular problem with the subsidized insurance system, as 70% of the total claims made come from this system [27]. The subsidized insurance scheme covers those individuals classified as poor according to a proxy means test (SISBEN); the scheme targets more vulnerable and poorer groups of population in Colombian society.

The three in-depth case studies will be purposively sampled using a range of criteria, including COVID-19 prevalence data, urbanity, health system design (e.g. mix of public and private providers) and technical capacity in public health. Data collection will involve remote and face-to-face interviews, documentary evidence and non-participant observations where possible (face-to-face aspect dependent on wider travel restrictions across Colombia at the time of phase 2 of the research). The case studies will focus on the planning and operational delivery of the response to COVID-19 in three major cities (Bogota, and two others chosen using the selection criteria above), and their governance relationships with the wider departments (states) in which they are situated and the national health system.

### Data collection

The aim of collecting qualitative data is to be build up a rich picture, or thick description [28], of the roles, responsibilities and relationships of different organizations making up the national and local health systems and, in particular, how the quality of relationships, including capacity constraints or deficits and innovative forms of integration to address capacity issues, are influencing responses to the COVID-19 pandemic.

Interviews (up to 30 per case study and up to 90 in total) will be conducted with stakeholders associated with change at different levels, including leaders of change planning at the national and local level, both within the health system and within municipal authorities; senior executives, operational managers and heads of relevant clinical departments, e.g. intensive care units, within provider organizations such as hospitals; other key stakeholders including insurance providers, regulatory authorities, professional associations and network-based organizations; and scientific or ‘expert’ advisers including official advisory groups.

Key themes explored in relation to each case study will include how different professions understand and participate in change processes; organizational processes including orientations toward collaboration with other organizations both within and beyond the health service in responding to COVID-19; factors influencing the capacity of healthcare organizations to respond to the pandemic, including availability of human resources, clinical space and equipment; inter-organizational relationships within the health system and with municipal organizations; their immediate priorities for improving the response to COVID-19 within their own organization and across the health system; and how the regulation and culture of the health system locally and nationally shapes the approach to, and implementation, of MSC in relation to COVID-19. The interviews will also explore the perceived resources that each health system has, including their technical capacity to respond to COVID-19, as well as their public health capacity to implement changes suggested by the study.

All study participants will be given an information sheet and provide informed consent. All discussions will be audio-recorded and informed by a topic guide. The topic guide for the interviews is provided (Additional file 1). The interviews will be transcribed in full by a professional transcription company. The researchers employed on the study will have experience undertaking qualitative research in Colombia, and they will be bilingual in Spanish and English to facilitate the production of reports and papers in both languages.

The interview data will be supplemented with analysis of documents (e.g. legal, financial and operational planning documents) at local and national level and non-participant observations where feasible (e.g. attending planning meetings) relating to the responses to COVID-19 will be conducted.

### Data analysis

For both phases 1 and 2, the interview transcripts, and other data such as notes of observations, will be analysed thematically using a combination of inductive and deductive coding [29]. Coding will be informed by the empirical data as it relates to the study objectives (inductive analysis) as well as themes in existing research on MSC and decision-making in crisis (deductive). Thematic analysis of data will involve collective and iterative coding by the research team: similar codes will be grouped together into themes, which will be prioritized in relation to addressing the driving research objectives and the potential contribution to existing understanding of the topic. The prioritized themes will be developed further through recursive coding of the transcripts. Any differences in interpretation of the coded data, and resulting themes, will be discussed among the research team and resolved through debate.

### Study activities by objective

By virtue of the need to deliver timely findings that can inform policy and practice, this study will be completed intensively over an 8-month period, under the leadership of the principal investigator and five full-time research assistants. The key activities, outputs and time allocation for their completion by study objective are summarized in Table 1.

**Table 1:** Summary of activities and products by objective

Objective	Activities	Products	Time
Ob1. To characterize, from the experiences of key actors of the health system at the national and local level, the strategies and organizational resources (including human resources and infrastructure) used to respond to the COVID-19 by each of the local health systems chosen for the study.	Finalize case study selection. The cases will include Bogotá and two other cities/departments with the highest prevalence of cases when initiating the study.	Summary of selected case study sites, including initial maps of health system roles, responsibilities and relationships and timelines of responses.	Month 1
	Conduct phase 1 stakeholder interviews (up to 30) at national level and in Bogotá. Collect documentary evidence on response to COVID-19	Qualitative dataset	Months 1–2
	Analysis of data collected from phase 1 of study data collection.	Paper submitted for peer-review publication and short summary shared of findings shared with health system stakeholders with the aim of informing decision-making.	Months 3–5
Ob2. To identify critical coordination issues or ‘bottlenecks’ that are constraining the capacity of the selected local health systems to respond to the pandemic as well as factors that facilitate the response.	Data collection for phase 2, covering all three case studies (up to 60 additional interviews and 90 in total). Collection of documentary evidence from all sites.	Qualitative dataset	Months 5–6
	Data analysis will focus on factors that constrain effective coordination in response to COVID-19. Analysis of information. This activity will take place from month 2. During the first 2 months, there will be a first analysis that we hope to socialize with the stakeholders involved. This first report, although exploratory, hopes to provide an input for actions in the short term.	See objective 3 products.	Months 7–8
Ob3. To analyse local examples of promising innovations and practices of coordination between actors that promote responsiveness to COVID-19 and have potential for scaling up more widely across health systems and/or integrated into healthcare guidelines or protocols.	Data collection for phase 2, covering all three case studies (up to 60 additional interviews and 90 in total). Collection of documentary evidence from all sites.	Qualitive dataset	Months 5–6
	Data analysis will focus on factors that enable effective coordination and promising innovations for ‘scale up’ in response to COVID-19.	Paper submitted for peer-review publication drawing on findings from phase 2 of the study (objectives 1–3). Accessible summary of findings will be shared with health system stakeholders with the aim of informing decision-making.	Months 7–8
Ob4. To establish mechanisms for sharing lessons and experiences of the crisis in a timely fashion with health system leaders and other key stakeholders to inform health service planning in relation to COVID-19 and future pandemics of national and international origin.	As part of the conduct and analysis of interviews, appropriate mechanisms sharing formative findings from the study will be identified.	Establishment of university-health system network to share study findings. Communication plan for sharing findings, including content, format and frequency of communication. Products aimed at stakeholders for sharing findings, including final report and lay summary, social media use, newsletters, project website, interactive guidance, webinars and other events.	Ongoing throughout study (months 1–8)

## Discussion

This study will speak to critical questions concerning the implementation of change under conditions that are far removed from planning ‘usual care’ and its improvement [30]. It will appraise the relevance of the concept of MSC in a context of ‘crisis’ decision-making and contribute to international lessons on improving the capacity and capabilities of health systems to respond to COVID-19 and future pandemics. In addition, the study will produce findings that aim to inform the planning of health services nationally and at a local health system level within Colombia on navigating MSC, recognizing the need to adapt, test and refine the theory in the context of a pandemic. To meet this purpose, data collection for this study will include assessing how best to share formative feedback from the research according to stakeholders’ preferences, and outputs from the study will include the building of a network with relevant communities of practice, short, accessible summaries of journal papers, and interactive guidance aimed at practitioner and policymaker audiences, the design and communication of which will draw on knowledge mobilization theory and experiences [19, 20].

As this study is being undertaken during the COVID-19 pandemic where physical distancing measures are in force, this raises a number of practical issues for its delivery through a virtual team. Firstly, virtual rather than face-to-face meetings among the research team will be necessary to deliver the study objectives. Collaborative software applications, such as Microsoft Teams, that allow videoconferencing and joint project work (e.g. virtual ‘brainstorming’ via synchronous population of documents) will be used to host team meetings.

Secondly, the research team will meet frequently for virtual meetings (e.g. twice per week) in order to set tasks and update on progress. Regular virtual meetings will be used to compensate for the lack of ‘informal’ alignment mechanisms that might otherwise take place through face-to-face interactions among the team, e.g. having corridor conversations in the management school or taking lunch or coffee together which is common practice on campus and part of the university’s established culture.

Thirdly, the full research team (the principal investigator and five full-time researchers) will not have met in person at the outset of the project and may not do depending on physical distancing measures, so ensuring a productive and positive working environment in the absence of trust and rapport cultivated through face-to-face engagement is a critical issue for the team. To compensate for this, the research team will experiment with a variety of activities to encourage conviviality at a distance, including icebreaking exercises, synchronous epistemic work (e.g. working on shared documents), attending online workshops or lectures together and other virtual social activities (e.g. drinks or food evenings).

Fourthly, interview and other forms of data will need to be collected virtually, unless physical distancing measures change during the course of the study. The interviews will be recorded using online software applications (e.g. Skype, Microsoft Teams or Zoom) and, where the interviewee prefers to undertake the interview by telephone, by using mobile phone apps for phone call recording. The conduct of virtual interviews may impact on the quality of the discussion (e.g. this type of communication may lack the opportunities for building rapport and the richness of a face-to-face interaction). However, the study will involve use of video-based interviews, where possible, in order to capture aspects of face-to-face interaction (e.g. mutual facial expressions), although the effect may be lessened as these are mediated by screen and the clarity of these will depend on the quality of the particular internet connection.

Another practical issue concerns the translation of interview and other qualitative data from Spanish to English, for the purposes of publication in peer-reviewed English and Spanish language journals. Quotes from the interviews and documents in Spanish chosen as relevant for reports and journals will be translated by the research team. Given that the team will be bilingual, researchers will be responsible for guaranteeing the fidelity and linguistic appropriateness of the translation to English.

Finally, we recognize that this study is taking place at a very challenging time for the planning and delivery of health services in Colombia and internationally. The study could add to this pressure as participation in the interviews will take up some of the respondents’ valuable time. However, the research team will do its best to minimize any inconvenience to participants by arranging to meet at a time suitable for them. Moreover, some participants may feel uncomfortable discussing the response to COVID-19 and its impact on health service planning and the delivery of care. As outlined in the study information sheet and consent form, potential participants will be free to withdraw from the study at any time and are encouraged to discuss any concerns they have before agreeing to take part. This protocol was written in accordance with Standards for Reporting Qualitative Research (SRQR) [31].

## Supplementary information

**Additional file 1.** Topic guide for stakeholder interviews.

**Additional file 2.** Ethics Approval Letter Universidad de los Andes.

**Additional file 3.** Funding Letter from Minciencias.

## Data Availability

For study updates follow @msc_covid19 or visit https://mscovid19.uniandes.edu.co/.

## Abbreviations

GNI: Gross National Income
LMIC: Low- and middle-income countries
MSC: Major system change
SISBEN: Sistema de Identificación de Potenciales Beneficiarios de Programas Sociales
SRQR: Standards for Reporting Qualitative Research
