# Supplementary material for: Qualitative analysis of the coordination of major system change within the Colombian health system in response to COVID-19: study protocol
Source: Implement Sci Commun. 2020 Sep 15;1:75. doi: 10.1186/s43058-020-00063-z (PMC7490777; doi:10.1186/s43058-020-00063-z)
Supplement: Supplementary file 1 — Additional file 1. Topic guide for stakeholder interviews. [file 43058_2020_63_MOESM1_ESM.docx]

**Topic guide for stakeholder interviews**

**Project title: Análisis de la coordinación de un cambio sistémico significativo en el sistema de salud colombiano como respuesta al COVID-19**

Major system change involves coordinated action by health care planners and providers to improve services across a whole health system (e.g. a metropolitan area such as Bogota). This case study focuses on the response of health planners and providers to COVID-19 within different local health sytems across Colombia. The purpose of these interviews is to establish stakeholders' priorities for health systems research on, and feedback approaches to support, the response to COVID-19. The bullet points are prompts, where needed.

1. What has been your organisation's experience of the pandemic?
   - Key challenges
   - Changes over time (national phase, LATAM & local prevalence, evidence availability)
   - How well prepared to respond (i.e preconditions of organisation)
2. What information have you used to respond to COVID-19? What is key, what is lacking?
   - Source (e.g. WHO, national, regional, internal, other health systems)
   - Type (audit data, research articles, clinical guidance, media reporting, protocols, legal rules)
3. Do you have adequate resources for responding to COVID-19?
   - Staff
   - Physical space
   - Equipment
   - Funding
   - Processes to support operations (including procurement / supply chain)

1. What plans do you have for changing capacity, if necessary?
   - Humanitarian plan actions
   - Provisional care units
   - Changing roles / responsibilities over time
2. Which other organisations are you working with in planning and delivering care (pre-existing and COVID-19 specific)?
   - Planning (CRUE – SDS – MSN - local and national government support)
   - Delivery (CRUE – Private institution – Private ambulance systems)
   - Public-private relationships (e.g. bed capacity/seconding staff/technical knowledge/ sharing equipment, e.g. Personal Protective Equipment (PPE), ventilators, oxygen)

1. Which relationships are working well, and where could coordination be improved?
   - Internal
   - External (including municipalites/local government and national government agencies)
   - Public-private relationships
2. What changes has your department or organisation made to address COVID-19?
   - Any 'innovative' practices, e.g. relationships, resource use, knowledge & skills?
3. What impact has COVID-19 had on your activities?
   - Direct in addressing coronavirus
   - Indirect impact on other services
4. What are your organization's priorities for improving the response to COVID-19?
5. What are the wider health system priorities across Bogotá (or Cali/Cartagena) for responding to COVID-19?
6. Following the pandemic, how do you consider that the future leadership, funding, planning and delivery of health and care services will be affected?
7. What feedback from this study would be most useful to you?
   - Types of information / Communication method / overcoming implementation issues, e.g. technical capacity in public health

*Close: Thank interviewee & request to add to stakeholder email list & ask for other interviewees*

SDS= Secretaría de Salud

CRUE= Centro Regulatorio de Urgencias y Emergencias
