# Supplementary material for: Qualitative analysis of the coordination of major system change within the Colombian health system in response to COVID-19: study protocol
Source: Implement Sci Commun. 2020 Sep 15;1:75. doi: 10.1186/s43058-020-00063-z (PMC7490777; doi:10.1186/s43058-020-00063-z)
Supplement: Supplementary file 2 — Additional file 2. Ethics Approval Letter Universidad de los Andes. [file 43058_2020_63_MOESM2_ESM.pdf]

**COMMITEE ON RESEARCH ETHICS**
**(1166- 2020)**

By means of this letter, the Committee on Research Ethics of the Universidad de los Andes certifies that the proposal **“Análisis de la coordinación de un cambio sistémico significativo en el sistema de salud colombiano como respuesta al COVID-19”** was reviewed and approved.

In accordance to the Colombian law, Resolución 8430 1993, this project classifies as “Riesgo Mínimo” or minimal risk. Professor Simon James William Tuner of the School of management at Universidad de los Andes presented the project to the Committee.

This certification is issued on this June 26th 2020

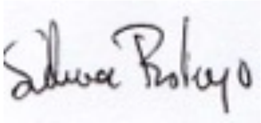

**SILVIA RESTREPO RESTREPO**
Committee Chair
