# Supplementary material for: Qualitative analysis of the coordination of major system change within the Colombian health system in response to COVID-19: study protocol
Source: Implement Sci Commun. 2020 Sep 15;1:75. doi: 10.1186/s43058-020-00063-z (PMC7490777; doi:10.1186/s43058-020-00063-z)
Supplement: Supplementary file 3 — Additional file 3. Funding Letter from Minciencias. [file 43058_2020_63_MOESM3_ESM.pdf]

El conocimiento es de todos | Minciencias

DIRGCS

Bogotá D.C., 24-06-2020

Doctor
SIMON JAMES WILLIAM TURNER.
Principal Investigator
Universidad de Los Andes.
s.turner@uniandes.edu.co
Bogotá D.C.

We certify that the research project "Analysis of the coordination of major system change within the Colombian health system in response to COVID-19: study protocol" was selected for funding under the call entitled "Invitation to present projects that contribute to solving current health problems related to the COVID-19 pandemic" (Invitación a presentar proyectos que contribuyan a la solución de problemáticas actuales de salud relacionadas con la pandemia de COVID-19).

www.minciencias.gov.co

This national call for research opened on the 25th of March, and the results were published on the 6th of April of 2020; 531 proposals were received in response to the call and 25 received funding. With the 25 funded, five projects that applied to the theme "Public Health Interventions related to the intervention regarding epidemiological risk" (Salud pública relacionada con la intervención frente a riesgos epidemiológicos) were funded and the project led by Dr. Simon Turner, Unidersidad de los Andes, received one of the highest scores from the peer review committee.

The study has been granted 569.989.187 Colombian pesos (equivalent to £ 122,322) and has been funded for the period 11 May 2020 to 11 January 2021.

Yours sincerely,

Luis Hernando Nieto Enciso.
Gestor Ciencia y Tecnología, Dirección de Generación de Conocimiento.

Elaboró: David A. Ribón O./Contratista/ Dirección Inteligencia de Recursos de la CTeI.
